# Genome Sequence of *Afipia felis* Strain 76713, Isolated in Hospital Water Using an Amoeba Co-Culture Procedure

**DOI:** 10.1128/genomeA.01367-14

**Published:** 2014-12-24

**Authors:** Samia Benamar, Bernard La Scola, Olivier Croce

**Affiliations:** Unité de Recherche sur les Maladies Infectieuses et Tropicales Emergentes, CNRS, UMR 6236, IRD 198, Faculté de Médecine, Aix-Marseille Université, Marseille, France

## Abstract

*Afipia felis* is a Gram-negative alphaproteobacterium originally described as the agent of cat-scratch disease (CSD). We sequenced the genome from a strain of *A. felis*, which was recovered from a hospital water sample using an amoebal co-culture procedure. It is composed of 3,989,646 bp, with a G+C content of 61.27% and encodes 4,068 protein-coding genes and 53 RNA genes.

## GENOME ANNOUNCEMENT

*Afipia felis* was first isolated from lymph nodes of patients with cat scratch disease (CSD) and was later seen as a cell contaminant (1, 2). The genus *Afipia* has been isolated from various types of patients, from an environmental water source, and from infected amoeba. *A. felis* 76713 was isolated in hospital water using an amoebal co-culture procedure in peptone yeast-extract glucose (PYG) medium (2). The 16S rRNA sequence of this strain was found to 99% identity using BLASTN with *A. felis* ATCC 53690^T^ (3). The member *A. felis* species are oxidase and nitrate reductase positive but catalase negative (4).

The genome was pyrosequenced on a 454 Roche Titanium sequencer using a 5 kb paired-end library (5). The 454 sequencing generated 406,562 reads assembled into contigs and scaffolds using Newbler v2.8 (Roche, 454 Life Sciences). Contigs obtained were combined together by Opera software v1.2 (6) combined with GapFiller v1.10 (7) to reduce the set. Finally, some manual refinements using CLC Genomics v5 software (CLC bio, Aarhus, Denmark) and homemade tools (http://bioinfomed.fr) improved the genome.

The draft genome of *A. felis* consists of 11 scaffolds of 322 contigs containing 3,936,701 bp and an estimated size including gaps of 3,989,646 bp. The G+C content of this genome is 61.27%, which is similar to other closed *A. felis* species (60.7%).

Noncoding genes and miscellaneous features were predicted using RNAmmer (8), ARAGORN (9), Rfam (10), PFAM (11). Open reading frames (ORFs) were predicted using Prodigal (12) and functional annotation was achieved using BLASTP against the GenBank database (13) and the Clusters of Orthologous Groups (COGs) database (14, 15).

The genome was shown to encode at least 53 RNA predicted genes including 3 rRNAs in a single operon and 50 tRNAs. 4,140 genes were also identified, representing a coding capacity of 3,369,783 bp (85.59% of the sequenced genome). Among these genes, 113 (2.72%) were founded as putative protein and 2,163 (52.24%) were assigned as hypothetical proteins. Moreover, 2,929 genes matched a least one sequence in the COGs database with BLASTP default parameters. Further, *A. felis* presents 25 genes annotated as resistance protein such as those related to copper resistance.

### Nucleotide sequence accession numbers.

The *A. felis* 76713 genome has been deposited at EMBL/EBI under the accession numbers CCAZ020000001 to CCAZ020000011.
